# Purely positive or discriminatorily positive? The development of two-factor attitudes toward lesbians and gay men scales

**DOI:** 10.3389/fpsyg.2023.1211282

**Published:** 2023-06-29

**Authors:** Lingfeng Guo, Shixin Fang, Hongbo Wen

**Affiliations:** ^1^Collaborative Innovation Center of Assessment for Basic Education Quality, Beijing Normal University, Beijing, China; ^2^Department of Human Development and Family Studies, The Pennsylvania State University, University Park, PA, United States

**Keywords:** attitudes toward lesbians/gay men, scales, reliability, validity, latent class analysis

## Abstract

Unidimensional bipolar scales based on prejudice against homosexuality neglect the effect of preference for heterosexuality on attitudes toward homosexuality. Additionally, the term “homosexuality” used in these scales may compromise their validity. The current study uses person-centered and variable-centered approaches to examine the structure and classes of attitudes toward lesbians and gay men. In Study 1, we developed the Two-factor Attitudes toward Lesbians and Gay Men Scales, which have acceptable reliability and validity. The results obtained through variable-centered approaches suggested that a model comprising two factors (prejudice against homosexuality and preference for heterosexuality) was ideal. In Study 2, we explored the classes of attitudes toward lesbians and gay men through latent class analysis. The results supported a model containing three classes (purely positive, discriminatorily positive, and negative). This study validates a two-factor structure of attitudes toward lesbians and gay men and distinguishes between purely positive and discriminatorily positive attitudes, providing an important reference for future research and interventions to promote public attitudes toward lesbians and gay men.

## Introduction

In previous decades, the legalized recognition of same-sex couples and the positive social climate related to LGBTQ+ individuals have transformed worldwide (Herek, 2006; Reczek, 2020). Nevertheless, LGBTQ+ populations still suffer from disproportionately high rates of mental health problems, such as depression, anxiety, suicide attempts, and substance abuse (Meyer and Dean, 1998; Plöderl and Tremblay, 2015; Russell and Fish, 2016; Ormiston and Williams, 2022). Prejudice based on sexual orientation has been recognized as a major predictor of the prevalence of disorders among sexual minorities (Meyer, 2003; Mongelli et al., 2019; Fulginiti et al., 2021). Attitudes toward lesbians and gay men merit attention, given that assessing such attitudes could improve the well-being of lesbians and gay men.

### Theory and measurement of attitudes toward homosexuality

A burgeoning body of research on attitudes toward homosexuality emerged in the 1970s (e.g., Davison and Wilson, 1973; Morris, 1973; Thompson and Fishburn, 1977; Garfinkle and Morin, 1978; Dressler, 1979). These studies were not explicitly informed by theoretical frameworks but theorized homosexuality as a psychopathology. A single item (e.g., “Most homosexual persons are mentally ill”) was used instead of a theory-based scale based on responses to a category variable (e.g., “yes,” “no,” or “cannot say”).

Subsequently, three theories of attitudes toward homosexuality were developed: the essentialist orientation of *homophobia*, the social constructivist orientation of the *old-fashioned prejudice*, and *modern prejudice against homosexuality* (the second and third are also known as *old-fashioned* and *modern homonegativity*; Morrison et al., 2009).

#### Homophobia

Weinberg (1972) first defined the term “homophobia” as hostility toward and an irritational fear of people identified as lesbian or gay. According to essentialist-oriented theory, homosexual behaviors violate the fundamental reproductive function of human sexual behaviors. Thus, homophobia is considered a human instinct (James, W. 1890). Numerous measurements based on the concept of homophobia have emerged in empirical research and practice (e.g., Hudson and Ricketts, 1980; Wright et al., 1999). However, psychosocial experiments have shown that homophobia is not a solely innate internal reaction elicited by personal traits (Herek, 2004; Inbar et al., 2012) but is more likely a result of power discrepancy related to sexuality (Herek, 2004, 2007; Russell and Bohan, 2006; Ray and Parkhill, 2021). As a result, research has increasingly focused on the influence of social and cultural factors on attitudes toward lesbians and gay men.

#### Old-fashioned homonegativity

Drawing on the commonalities of sexism and racism, researchers proposed the notion of homosexism (Lehne, 1976). In comparison with homophobia, homosexism more strongly emphasizes the sociocultural background underlying the formation of sexuality-related stigmas. Hudson and Ricketts (1980) used the term “homonegativism” to combine the content referred to by “homophobia” and “homosexism.” In this vein, researchers came to use “homonegativity” to refer to negative attitudes toward lesbians and gay men (Lingiardi et al., 2016; Falgares et al., 2022). The Attitudes Toward Lesbians and Gay Men Scale, developed by Herek (1988), is currently one of the most extensively applied and revised measures of attitudes toward homosexuality.

#### Modern homonegativity

As research on prejudice intensified, researchers proposed two types of prejudice: blatant prejudice (old-fashioned prejudice) and subtle prejudice (modern prejudice) (Swim et al., 1995; Pedersen and Walker, 1997). Inspired by the modern prejudice theory, Morrison and Morrison (2002) suggested that prejudice against lesbians and gay men has transformed from old-fashioned homonegativity to modern homonegativity. They also proposed that old-fashioned and modern homonegativity are independent structures. Old-fashioned homonegativity was defined as a misunderstanding of homosexuality based on traditional religious and moral beliefs, but this definition has become outdated. Meanwhile, modern homonegativity was more closely related to neglecting rights, denying discrimination, and generalizing inter-group differences. As a result, Morrison and Morrison (2002) developed the Modern Homonegativity Scale, which contains 13 widely employed items (e.g., Morrison et al., 2005, 2009; Romero et al., 2015). However, Lottes and Grollman (2010) tested the modern homonegativity hypothesis, and the results did not support the assumption that old-fashioned and modern homonegativity are independent constructs. Moreover, participants reported similar levels of old-fashioned and modern homonegativity toward gay men and lesbians.

### Problems with theories and research on attitudes toward homosexuality

Homophobia, old-fashioned homonegativity, and modern homonegativity focus on negative attitudes toward lesbians and gay men. These theories take such attitudes as unidimensional structures and suppose that individuals’ attitudes are presented through cognition, feelings, and behavioral tendencies. As a result, existing measurements using the above theories as frameworks use item composite scores with a unidimensional bipolar scale (extreme positive to extreme negative) to interpret scores. Positive and negative attitudes are considered mutually exclusive, which implies that attitudes toward lesbians and gay men cannot be both positive and negative. As such, previous research generally categorized attitudes toward lesbians and gay men into positive and negative classes (Larsen et al., 1980; Kite and Deaux, 1986; Herek, 1988; Morrison et al., 1999; Morrison and Morrison, 2002).

However, positive and negative attitudes toward lesbians and gay men could be independent. For example, Jackman (2020) found that the majority of a heterosexual sample held conflicting attitudes toward anti-gay laws in Barbados and sexual activity between people of the same sex. Clausell and Fiske (2005) also found that college students simultaneously had positive and negative stereotypes of gay men. Through qualitative research methods, Wang (2011) found that some lesbians and gay men also had contradictory attitudes toward themselves.

Additionally, the types of attitudes toward lesbians and gay men could fit into more than two classes (i.e., positive and negative). Guo (2023) identified that the dichotomy of positive and negative limits researchers’ ability to distinguish between purely positive and discriminatorily positive attitudes, which differ from one another. Compared to the purely positive class, the discriminatorily positive class entails conditional, selective, and incomplete positivity based on a heteronormative culture. In line with the manifestation of tepid homophobia and veiled homophobia in a novel framework analyzing homophobia (Lyonga, 2021), people holding discriminatorily positive attitudes may not oppose the practice of homosexuality but may be against granting lesbian women and gay men the same rights as heterosexual people.

Lesbians and gay men may also suffer from subtle forms of prejudice masked by a guise in daily scenarios. For example, some individuals could accept others’ homosexuality but not their children’s (Tan et al., 2012; Liang, 2015), or they might have positive attitudes only toward “good” lesbians and gay men who meet their expectations based on heteronormative norms (Bai et al., 2013). Common views of discriminatorily positive attitudes include “homosexuality is normal, but I hope they will not be *[sic]* conspicuous” and “if homosexuals could become heterosexuals, they would be happier.” The above “positive” attitudes may be well-intentioned to a certain extent, but they reinforce power and status discrepancies between heterosexuals and non-heterosexuals.

### The two-factor and three-class model of attitudes toward homosexuality

Inspired by postmodern feminist psychology, Guo (2023) deconstructed and reconstructed the theory of attitudes toward homosexuality from a sexual-orientation-diverse perspective rather than a single perspective (i.e., *prejudice against homosexuality*). In this vein, Guo (2023) developed a two-factor and three-class model of attitudes toward homosexuality and the Two-factor Attitudes toward Homosexuality Scale (TAHS).

Theories on *prejudice against homosexuality* have highlighted the importance of the binary opposition between homosexuality and heterosexuality. *Preference for heterosexuality* is established based on an individual’s conscious or unconscious admiration for and reinforcement of heteronormativity. As such, when *prejudice against homosexuality* (Factor 1) is non-existent or low, attitudes should be identified as positive; when the level of Factor 1 is high, attitudes should be negative. Likewise, when there is no or a low *preference for heterosexuality* (Factor 2), the corresponding attitude is considered positive, while a high level of Factor 2 is considered negative. An inconsistency in attitudes toward Factor 1 and Factor 2 reflects an ambivalent attitude. This hypothesis of a two-factor structure of attitudes toward homosexuality can reconcile the problems posed by the coexistence of positive and negative attitudes.

Additionally, the combination of *prejudice against homosexuality* and *preference for heterosexuality* theoretically results in three classes of attitudes toward homosexuality: purely positive, discriminatorily positive, and negative. This hypothesis of a three-class model of attitudes toward homosexuality can help researchers distinguish between purely positive and discriminatorily positive classes. Examples of the three combinations are as follows:

1. Positive in Factor 1 + positive in Factor 2 = purely positive attitude
2. Positive in Factor 1 + negative in Factor 2 = discriminatorily positive attitude
3. Negative in Factor 1 + negative in Factor 2 = negative attitude

Note that the two-factor and three-class model focuses on attitudes toward homosexuality instead of the specific sub-groups of lesbians and gay men. The term “homosexuality” used in measures might cause measurement bias. Such a use of “homosexuality” led to two main issues in previous studies.

Firstly, the results from such a measurement hardly explain whether the measurement represents attitudes toward homosexuality or attitudes toward gay men. There are several reasons for this. For one, when presented with the term “homosexuals,” some participants might consider gay men the target group (Black and Stevenson, 1984; Herek, 1984). Similarly, qualitative research has indicated that lesbians are easily neglected or replaced by gay men in some homosexual rights movements for historical and social reasons (Cheng, 2018). Additionally, descriptions of gay men are more common than those of lesbians in literary works, artistic works, and historical records (Zhang, 2007), which may cause the concept of lesbians to be absent from some people’s concept of ‘homosexuality (Ruan and Tsai, 1987; Cheng, 2018).

Secondly, even if the term “homosexual” is considered to represent both lesbians and gay men, individuals’ attitudes toward lesbians and gay men are different—normally, attitudes toward lesbians are more positive than those toward gay men (Poteat and Espelage, 2005; Kosciw et al., 2009; Zhu et al., 2012). As such, if a participant has positive attitudes toward lesbians and negative attitudes toward gay men, the use of the term “homosexuality” limits participants’ ability to reflect on whether their attitudes toward lesbians differ from their attitudes toward gay men.

We conducted two studies using variable-centered and person-centered approaches to validate whether the two-factor and three-class model of attitudes toward homosexuality is suitable for assessing attitudes toward lesbians and gay men. Additionally, we developed two new scales—the Two-factor Attitudes toward Lesbians and Gay Men Scales (TAHS-L and TAHS-G)—to evaluate the applicability of this model and the interpretability of the results of people’s attitudes.

## Study 1: the structure of attitudes toward lesbians and gay men

### Method

#### Participants

We recruited participants through the Wenjuan Network platform. The Wenjuan Network platform is a relatively mature and reliable questionnaire data collection platform in China that is widely used by researchers. This platform can provide participants with response time and IP addresses to eliminate invalid data or duplicate responses, and participants cannot submit data until they complete all items, meaning there are no missing data.

After data associated with abnormal time-consumption were deleted, data from 2,673 participants (1,339 heterosexual women, 532 heterosexual men, 545 non-heterosexual women, and 257 non-heterosexual men) were used in exploratory factor analysis (EFA) (first stage). Furthermore, data from 2,720 participants (1,359 heterosexual women, 541 heterosexual men, 559 non-heterosexual women, and 261 non-heterosexual men) were used in confirmatory factor analysis (CFA), reliability analysis, and measurement invariance (second stage). Finally, data from 361 participants (88 heterosexual women, 86 heterosexual men, 122 non-heterosexual women, and 65 non-heterosexual men) were used in the convergent validity test (third stage). Participants’ ages ranged from 18 to 40. More demographic characteristics of the participants are presented in Table 1. The data in the current study were different from the data used to develop the TAHS (Guo 2023).

**Table 1:** Participants’ demographic characteristics.

Variable	First stage	Second stage	Third stage
	(*N* = 2,673)	(*N* = 2,720)	(*N* = 361)
Sex, *N* (%)			
Male	789 (29.5%)	802 (29.5%)	151 (41.8%)
Female	1,884 (70.5%)	1,918 (70.5%)	210 (58.2%)
Gender identity, *N* (%)			
Man	818 (30.6%)	831 (30.6%)	146 (40.4%)
Women	1,833 (68.6%)	1,867 (68.6%)	200 (55.4%)
Other	22 (0.8%)	22 (0.8%)	15 (4.2%)
Sexual orientation, *N* (%)			
Heterosexual	1,871 (70.0%)	1,900 (69.9%)	174 (48.2%)
Homosexual	275 (10.3%)	278 (10.2%)	90 (24.9%)
Bisexual	326 (12.2%)	335 (12.3%)	59 (16.3%)
Questioning	174 (6.5%)	180 (6.6%)	27 (7.5%)
Other	27 (1.0%)	27 (1.0%)	11 (3.0%)
Hometown, *N* (%)			
City	1,216 (45.5%)	1,240 (45.6%)	226 (622.6%)
Town	816 (30.3%)	831 (30.6%)	104 (28.8%)
Countryside	641 (24.5%)	649 (23.9%)	31 (8.6%)
Degree, *N* (%)			
Secondary school or below	245 (9.2%)	251 (9.2%)	24 (6.6%)
Bachelor’s degree	1,345 (50.3%)	1,373 (50.5%)	188 (52.1%)
Master’s degree or above	1,083 (40.5%)	1,096 (40.3%)	149 (41.3%)

#### Measures

Based on the TAHS developed by Guo (2023), we developed the TAHS-L and TAHS-G, each of which contains 12 items. The TAHS-L and TAHS-G were developed and tested in the Chinese language. Participants responded to each item using a 4-point Likert scale (1 = “strongly disagree,” 2 = “relatively disagree,” 3 = “relatively agree,” and 4 = “strongly agree”). After several items were reverse-scored, the highest possible score was 48. Higher scores indicated more positive attitudes toward lesbians and gay men.

We assessed the convergent validity using the Pearson’s coefficients between the scores of TAHS-L/G and the Attitudes toward Homosexuality Scale (AHS). The AHS utilizes a 5-point Likert scale and comprises 10 items for attitudes toward lesbians and 10 items for attitudes toward gay men. Higher scores indicate more positive attitudes toward homosexuality. The Cronbach’s *α* of AHS-Lesbian and AHS-Gay were 0.910 and 0.905, according to Yu et al. (2010). Participants answered all items in a random order to avoid the sequence effect of responses.

### Data analysis

We utilized EFA to extract common factors of the TAHS-L/G and assessed the TAHS-L/G’s construct validity through CFA. The TAHS-L/G’s convergent validity was tested based on the correlation coefficient between the TAHS-L/G and existing scales. The reliability tests included the Pearson correlation coefficients between item scores and total scores, Cronbach’s *α*, and split-half reliability. We ran multi-group CFAs and DIFFTEST to assess the measurement invariance of sex. All analyses were conducted in SPSS 22.0 and Mplus 8.

### Results

#### Exploratory factor analysis

We performed the Kaiser-Meyer-Olkin test, the Bartlett sphericity test, and item correlation analysis. The results showed that most correlation coefficients exceeded 0.3 (*p* < 0.001). Specifically, the Kaiser-Meyer-Olkin test value was 0.929 for the TAHS-L and 0.935 for the TAHS-G (>0.5). Furthermore, the measures of sampling adequacy for each item exceeded 0.5 on the diagonal of the image correlation matrix. Therefore, the data met the prerequisites for EFA.

We conducted an EFA on 12 items of the TAHS-L and TAHS-G and extracted one to four factors. Tables 2, 3 present the statistics for the number of parameters (k), chi-square value (*χ*^2^), degrees of freedom (*df*), and value of p. The results show that the chi-square value decreased the most when two factors and one factor were compared. When three factors and two factors or four factors and three factors were compared, the chi-square value decreased to a lesser extent. The results suggest that the two-factor model is the best option for the TAHS-L and TAHS-G.

**Table 2:** Model comparisons for the TAHS-L.

		*χ* ^2^	*df*	*p*
**Model**	**k**			
one-factor	12	1149.820	54	0.000
two-factor	23	449.233	43	0.000
three-factor	33	248.211	33	0.000
four-factor	42	66.329	24	0.000
**Model comparisons**				
one-factor against two-factor		535.372	11	0.000
two-factor against three-factor		174.212	10	0.000
three-factor against four-factor		140.058	9	0.000

**Table 3:** Model comparisons for the TAHS-G.

		*χ* ^2^	*df*	*p*
**Model**	**k**			
one-factor	12	1599.392	54	0.000
two-factor	23	661.275	43	0.000
three-factor	33	163.526	33	0.000
four-factor	42	69.383	24	0.000
**Model comparisons**				
one-factor against two-factor		154.732	691.538	11
two-factor against three-factor		71.601	358.902	10
three-factor against four-factor		19.741	75.929	9

Since we used a 4-point Likert scale, we also used the weighted least squares with adjusted mean and variance (WLSMV) method, which can handle the category variables required to estimate the factors. Principal components analysis revealed two common factors with eigenvalues greater than 1. As a result, we used the WLSMV estimation on the two-factor model; the results yielded through this process are presented in Table 4.

**Table 4:** Fit statistics of the two-factor model of the TAHS-L/G.

	TLI	CFI	RMSEA (90% CI)	SRMR
TAHS-L	0.984	0.990	0.059 [0.055, 0.064]	0.029
TAHS-G	0.981	0.988	0.073 [0.068, 0.078]	0.029

The evaluation indexes for the model included root mean square error of approximation (RMSEA), standardized residual root mean square (SRMR), comparative fit index (CFI), and Tucker-Lewis index (TLI). Generally, smaller RMSEA and SRMR values indicate better model fit. The closer the CFI and TLI values are to 1, the better the model’s fit.

Due to cross-loadings and inter-factor correlations, we adopted the direct oblimin rotation method. For the four-factor model of the TAHS-L, the loadings of most variables on the third and fourth potential factors are relatively small. For the three-factor model of the TAHS-L, the loadings of most variables on the third factor are relatively small. For the two-factor, all variables’ factor loadings are greater than 0.500 on a single potential factor and lower than 0.300 on another factor. Similarly, for the four-factor model of the TAHS-G, the loadings of most variables on the third and fourth potential factors are relatively small. For the three-factor model of the TAHS-G, the loadings of most variables on the second and third factors are relatively small. For the two-factor of TAHS-G, all variables’ factor loadings are greater than 0.500 on a single potential factor and lower than 0.300 on another factor. Therefore, it was determined that the two-factor models of both the TAHS-L and TAHS-G are more suitable than the three-factor and four-factor models. Based on the items’ respective meanings, we named Factor 1 “prejudice against homosexuality” and Factor 2 “*preference for heterosexuality*.” Table 5 presents the items and factor loadings of the two-factor model of the TAHS-L/G.

**Table 5:** Items and factor loadings of the two-factor model of the TAHS-L/G.

Scale	Item number	Item description	Factor 1	Factor 2
TAHS-L	L-x3	Lesbians should be condemned.	0.874	
	L-x5	Lesbianism is an inferior form of sexuality.	0.861	
	L-x4	Lesbians are sinners.	0.839	
	L-x6	Lesbians are immoral.	0.811	
	L-x2	Lesbians should have equal employment opportunities.	0.714	
	L-x1	Lesbians should be treated fairly in society.	0.703	
	L-x10	It is better for lesbians to conceal their sexual orientation.		0.808
	L-x9	If lesbians want to be respected, they should remain low-key and avoid being conspicuous.		0.757
	L-x8	If lesbians could become heterosexuals, they would be happier.		0.738
	L-x7	I cannot accept my relatives being lesbians.		0.540
	L-x11	I try to avoid being friends with lesbians.		0.463
	L-x12	I can get along with lesbians comfortably.		0.442
TAHS-G	G-x3	Gay men should be condemned.	0.824	
	G-x4	Gay men are sinners.	0.813	
	G-x6	Gay men are immoral.	0.809	
	G-x5	Being a gay man is an inferior form of sexuality.	0.807	
	G-x2	Gay men should have equal employment opportunities.	0.805	
	G-x1	Gay men should be treated fairly in society.	0.748	
	G-x10	It is better for gay men to conceal their sexual orientation.		0.821
	G-x9	If gay men want to be respected, they should remain low-key and avoid being conspicuous.		0.753
	G-x8	If gay men could become heterosexuals, they would be happier.		0.734
	G-x7	I cannot accept my relatives being gay men.		0.550
	G-x11	I try to avoid being friends with gay men.		0.450
	G-x12	I can get along with gay men comfortably.		0.438

### Confirmatory factor analysis

The mean, standard deviation, skewness, and kurtosis coefficients of each item are shown in Table 6. The absolute skewness coefficient values of all items ranged from 0.481 to 3.848, and the absolute kurtosis coefficient values ranged from 0.276 to 15.925.

**Table 6:** Descriptive statistics of each item.

Item	*M*	SD	Skewness coefficient	Kurtosis coefficient
L-x1	3.65	0.700	−2.135	4.094
L-x2	3.76	0.606	−2.850	8.274
L-x3	3.83	0.515	−3.695	14.787
L-x4	3.82	0.528	−3.569	13.678
L-x5	3.84	0.505	−3.848	15.925
L-x6	3.80	0.570	−3.338	11.696
L-x7	3.42	0.963	−1.525	1.020
L-x8	3.31	0.934	−1.177	0.276
L-x9	3.14	1.003	−0.839	−0.521
L-x10	3.11	0.930	−0.713	−0.504
L-x11	3.62	0.782	−2.166	3.855
L-x12	2.94	0.964	−0.481	−0.811
G-x1	3.51	0.811	−1.675	0.094
G-x2	3.65	0.720	−2.276	4.694
G-x3	3.76	0.624	−3.048	9.340
G-x4	3.76	0.643	−3.039	8.911
G-x5	3.78	0.595	−3.182	10.379
G-x6	3.73	0.667	−2.804	7.638
G-x7	3.34	1.018	−1.334	0.375
G-x8	3.27	0.951	−1.129	0.173
G-x9	3.00	1.032	−0.619	−0.862
G-x10	3.06	0.958	−0.688	−0.572
G-x11	3.52	0.889	−1.818	2.158
G-x12	2.76	1.011	−0.291	−1.030

We conducted a CFA on the two-factor model, which contains two latent variables and 12 measurement indicators with 78 pieces of information (p*(p + 1)/2). There are 25 parameters to estimate, including 12 factor loadings, one covariance between the two factors, and the error variance of 12 items (78–25 = 53 (*df*)). Furthermore, there are more than three items for each factor without relevant error. Therefore, the model conforms to the rules of CFA model identification.

**Figure 1:**
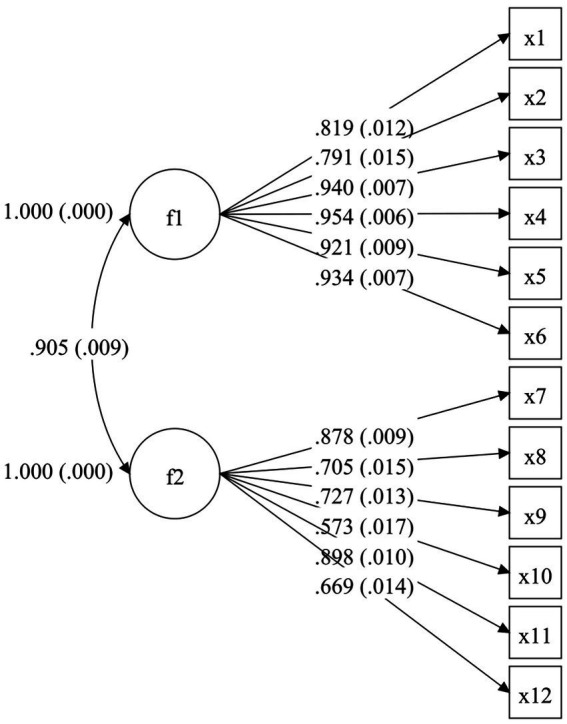
CFA path diagram (TAHS-L). Factor 1 refers to prejudice against homosexuality, and Factor 2 refers to preference for heterosexuality.

**Figure 2:**
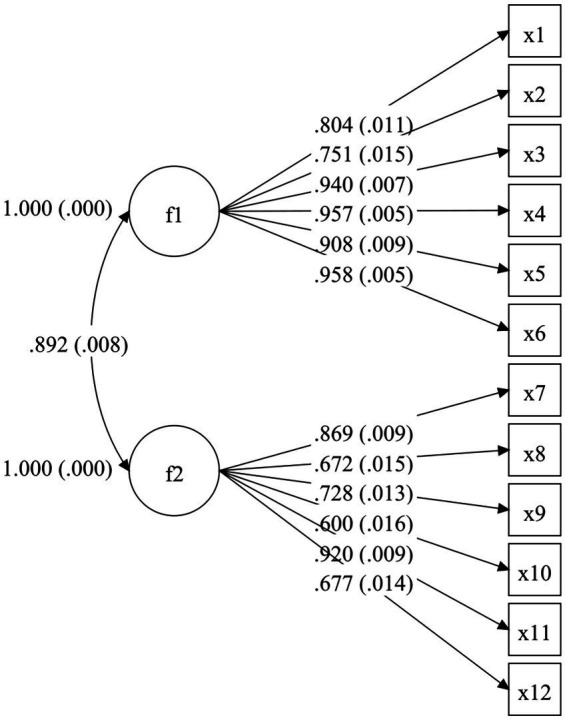
CFA path diagram (TAHS-G).

The WLSMV estimation yielded the following results: (1) TAHS-L: *χ*^2^ = 874.316，*df* = 53，*χ*^2^/*df* = 16.497， RMSEA (90% CI) = 0.075 [0.071, 0.080]， CFI = 0.980，TLI = 0.975， SRMR = 0.035; (2) TAHS-G: *χ*^2^ = 1103.453, *df* = 53, *χ*^2^/*df* = 20.820, RMSEA (90% CI) = 0.085 [0.081, 0.090], CFI = 0.979, TLI = 0.974, SRMR = 0.037. These results show that the two-factor models of TAHS-L and TAHS-G fit well. Figures 1, 2 present the path diagrams of the TAHS-L and TAHS-G.

### Reliability analysis

1. TAHS-L: The Pearson correlation coefficients between each item score and the total score were between 0.560 and 0.799. The coefficients between total scores on Factor 1 and its six constituent items ranged between 0.722 and 0.847, and those between total scores on Factor 2 and its six constituent items ranged between 0.643 and 0.789. The Cronbach’s *α* for the full scale was 0.885 (0.877 for Factor 1 and 0.810 for Factor 2). The split-half reliability of the scale was 0.833 (0.742 for Factor 1 and 0.707 for Factor 2).
2. TAHS-G: The Pearson correlation coefficients between each item score and the total score were between 0.579 and 0.805. The coefficients between the total scores on Factor 1 and its six constituent items ranged between 0.728 and 0.874, and those between the total scores on Factor 2 and its six constituent items ranged between 0.663 and 0.795. The Cronbach’s *α* for the full scale was 0.885 (0.897 for Factor 1 and 0.896 for Factor 2). The split-half reliability of the scale was 0.844 (0.765 for Factor 1 and 0.717 for Factor 2). These results suggest that the TAHS-L and TAHS-G have acceptable reliability.

### Convergent validity

We found that the TAHS-L has a significant positive correlation with the AHS-L, *r* (361) = 0.806, *p* < 0.001. The scores of the *prejudice against homosexuality* factor of the TAHS-L had a significant positive correlation with the AHS-L, *r* (361) = 0.786, *p* < 0.001, and the scores of the *preference for heterosexuality* factor of the TAHS-L had a significant positive correlation with the AHS-L, *r* (361) = 0.849, *p* < 0.001. Likewise, the TAHS-G had a significant positive correlation with the AHS-G, *r* (361) = 0.940, *p* < 0.001. The scores of the *prejudice against homosexuality* factor of the TAHS-G had a significant positive correlation with the AHS-G, *r* (361) = 0.886, *p* < 0.001, and the scores of the *preference for heterosexuality* factor of the TAHS-G had a significant positive correlation with the AHS-G, *r* (361) = 0.875, *p* < 0.001.

### Measurement invariance of sex

Because the chi-square test is easily affected by sample size, and since we used a 4-point Likert scale and, thus, the variable measured using this scale is considered an ordinal variable, we used the WLSMV method for parameter estimation, as well as several fitting indexes, including the Tucker-Lewis Index (TLI), comparative fit index (CFI), root mean square error of approximation (RMSEA), and standardized root mean square residual (SRMR). When comparing nested models, we used the DIFFTEST, which can handle ordinal variables (Li and Liu, 2011). To compare multi-group CFAs and nested models, we used four models: the Configural Invariance Model, Metric Invariance Model, Threshold Invariance Model, and Residual Variance Invariance Model.

1. CFA in a Single Group. Table 7 presents the CFA results for each group. The results suggest that each model fits well and that it is acceptable to use these models as baseline models for assessing measurement invariance in subsequent analyses.
2. Measurement Invariance of the TAHS-L. We used the male group as the reference group. As shown in Table 8, the Configural Invariance Model (M1) had good fit. The equality of the unstandardized item factor loadings between groups was then examined in M1. The DIFFTEST shows that the Metric Invariance Model (M2) fitted significantly worse than M1 (*p* < 0.01). The modification indices suggested a localized misfit for the constrained loading of Item 10. After its loading between groups was freed, the Partial Metric Invariance Model (M2B) did not fit significantly worse than M1 (*p* = 0.07).

**Table 7:** Fit statistics of each group.

Scale	Group	*N*	*χ* ^2^	*df*	TLI	CFI	RMSEA (90% CI)	SRMR
TAHS-L	Male	802	336.643	53	0.981	0.985	0.083 [0.073,0.090]	0.036
	Female	1,918	640.944	53	0.962	0.969	0.076 [0.071,0.081]	0.043
TAHS-G	Male	802	466.604	53	0.980	0.984	0.099 [0.091,0.107]	0.040
	Female	1918	701.030	53	0.958	0.966	0.080 [0.075,0.085]	0.043

**Table 8:** Fit statistics of sex invariance (TAHS-L).

Model	*χ* ^2^	*df*	TLI	CFI	RMSEA (90% CI)	SRMR	DIFFTEST
M1	994.969	106	0.970	0.976	0.079 [0.074,0.083]	0.041	–
M2	719.480	116	0.982	0.984	0.062 [0.058,0.066]	0.044	32.15^*^
M2B	720.642	115	0.981	0.984	0.062 [0.058,0.067]	0.042	15.98
M3	774.284	149	0.985	0.983	0.056 [0.052,0.059]	0.042	69.98^*^
M3B	746.240	145	0.985	0.984	0.055 [0.051,0.059]	0.042	35.43
M4	872.004	134	0.981	0.980	0.064 [0.060,0.068]	0.042	12.57

^*^Indicates that *p* < 0.05 and that the model fitted significantly worse than the former model.

The equality of the unstandardized item thresholds across groups was then examined in a Threshold Invariance Model (M3). The DIFFTEST shows M3 fitted significantly worse than M2B (*p* < 0.01), and the modification indices suggest that Items 8 and 9 were the largest sources of the misfit. After the thresholds of Items 8 and 9 were freed, the partial Threshold Invariance Model (M3B) did not fit significantly worse than M2B (*p* = 0.23). Finally, the equality of the unstandardized residual variances across groups was examined in a Residual Variance Invariance Model (M4). The model comparison at this step proceeded backward. M4 had good fit, and it did not fit significantly worse than M3B (*p* = 0.32).

1. Measurement Invariance of the TAHS-G. We considered the male group as the reference group. As shown in Table 9, the Configural Invariance Model (M1) had good fit. The equality of the unstandardized item factor loadings between groups was then examined in M1. The DIFFTEST shows that the Metric Invariance Model (M2) fitted significantly worse than M1 (*p* < 0.01). The modification indices suggest a localized misfit for the constrained loading of Item 8. After this item’s loading between groups was freed, the Partial Metric Invariance Model (M2B) did not fit significantly worse than M1 (*p* = 0.15).

**Table 9:** Fit statistics of sex invariance (TAHS-G).

Model	*χ* ^2^	*df*	TLI	CFI	RMSEA (90% CI)	SRMR	DIFFTEST
M1	1185.314	106	0.970	0.976	0.087 [0.082,0.091]	0.042	–
M2	767.302	116	0.983	0.985	0.064 [0.060,0.069]	0.045	25.91^*^
M2B	778.715	115	0.983	0.985	0.065 [0.061,0.070]	0.044	13.20
M3	871.717	149	0.986	0.984	0.060 [0.056,0.064]	0.045	106.63^*^
M3B	816.092	141	0.986	0.985	0.059 [0.055,0.063]	0.044	36.59
M4	1057.499	130	0.979	0.979	0.072 [0.068,0.077]	0.043	17.83

^*^Indicates that *p* < 0.05 and that the model fitted significantly worse than the former model.

The equality of the unstandardized item thresholds across groups was then examined in a Threshold Invariance Model (M3). The DIFFTEST shows M3 fitted significantly worse than M2B (*p* < 0.01), and the modification indices suggest that Items 7, 10, and 12 were the largest sources of misfit. After the thresholds of Items 7, 10, and 12 were freed, the Partial Threshold Invariance Model (M3B) did not fit significantly worse than M2B (*p* = 0.08). Finally, the equality of the unstandardized residual variances across groups was examined in a Residual Variance Invariance Model (M4). The model comparison at this step proceeded backward. M4 had good fit, and it did not fit significantly worse than M3B (*p* = 0.09).

In conclusion, the above analyses show that the partial measurement invariance of both the TAHS-L and TAHS-G was obtained across males and females. That is, the relationships of the items with the latent factor of independent living were equivalent between males and females.

## Study 2: the classes of attitudes toward lesbians and gay men

### Method

#### Participants and measures

In this study, we used TAHS-L/G, and the data were the same as those used in the second stage (*N* = 2,720) of Study 1.

### Data analysis

We conducted latent class analysis in Mplus 8—a person-centered approach—to test the validity of the three-class model. We used the maximum likelihood method to estimate the parameters, and we used the maximum expectation method in the iterative process. The evaluation indexes of the model included the Akaike information criterion, the Bayesian information criterion, and the adjusted Bayesian information criterion. Generally, smaller values for these indexes indicate better model fit. We evaluated the classification accuracy with the entropy index, and we used the Lo–Mendell–Rubin test and the bootstrapped likelihood ratio test to compare fit differences among the latent class models. If the value of p of an index indicated statistical significance, we concluded that the k-class model fitted significantly better than the k-1 class model (Wang, 2014).

### Results

Taking the 12 scale items as indicators, we converted the 2,720 responses provided on the 4-point scale into values between 0 and 1. “Relatively disagree” and “strongly disagree” responses were recorded as 0 (which represents a negative tendency), and “relatively agree” and “strongly agree” responses were recorded as 1 (which represents a positive tendency). We performed latent class analyses, with each analysis considering between one and four classes.

Tables 10, 11 display the fit statistics of the TAHS-L and TAHS-G, respectively, which are demonstrated through comparisons of the different models’ fit statistics. Critically, the Akaike information criterion, Bayesian information criterion, and adjusted Bayesian information criterion values of the three-class model decreased the most significantly. These results support the use of the three-class model for both the TAHS-L and TAHS-G.

**Table 10:** Fit statistics of four models (TAHS-L).

Number of classes	k	AIC	BIC	aBIC	Entropy	LMR	BLRT	Class probability
1	12	22095.27	22166.17	22128.05	–	–	–	1
2	25	18694.42	18842.13	18762.70	0.91	<0.001	<0.001	0.857/0.143
3	38	18074.81	18299.31	18178.59	0.86	<0.001	<0.001	0.204/0.032/0.764
4	51	17917.56	18218.89	18056.84	0.76	0.012	<0.001	0.032/0.138/0.171/0.659

k refers to the number of free parameters.

**Table 11:** Fit statistics of four models (TAHS-G).

Number of classes	k	AIC	BIC	aBIC	Entropy	LMR	BLRT	Class probability
1	12	26362.17	25433.07	25394.94	–	–	–	1
2	25	20978.09	21122.80	21043.36	0.921	<0.001	<0.001	0.156/0.844
3	38	20242.94	20467.46	20346.72	0.863	<0.001	<0.001	0.749/0.198/0.053
4	51	20033.31	20334.63	20172.59	0.847	0.005	<0.001	0.082/0.722/0.056/0.139

**Figure 3:**
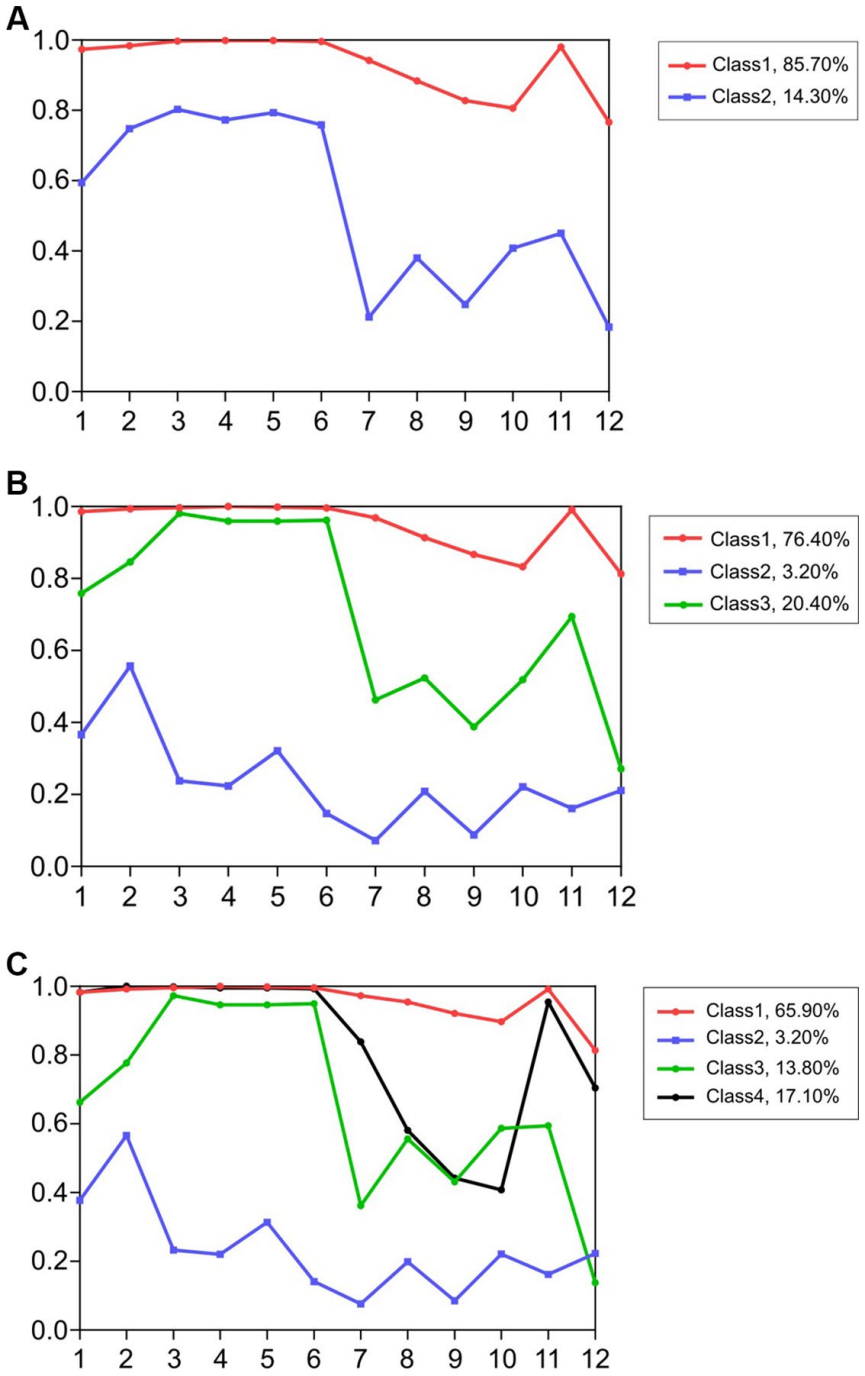
**(A)** Conditional probability diagrams of the two-class model (TAHS-L). **(B)** Conditional probability diagrams of the three-class model (TAHS-L). **(C)** Conditional probability diagrams of the four-class model (TAHS-L).

Figures 3A–C display the conditional probability diagrams of two to four classes of attitudes toward lesbians.

*Conditional probability diagrams of two to four classes. The abscissa represents the item, and the ordinate represents the response probability (*i.e.*, the probability of each participant’s positive tendency on each item)*.

In the two-class model (Figure 3A), the scale could divide participants into two classes, namely a positive attitude group (red line) and a negative attitude group (blue line). In the three-class model (Figure 3B), the conditional probabilities of the first class (red line) and second class (green line) for the *prejudice toward homosexuality* factor (the items on the abscissa were 1–6) were very similar; the conditional probabilities of the second class (green line) and the third class (blue line) for the *preference for heterosexuality* factor (the items on the abscissa were 7–12) were also very similar. In the four-class model (Figure 3C), the fourth class (black line) appeared in the conditional probabilities, whereas the third class was more explanatory than the other classes.

Therefore, we selected the three-class model, combined with the various fit indexes, the model diagrams, and the interpretability of each class as the most suitable model. According to the responses for all items of the two factors in the three-class model, the first class’s scores on both factors were very high, which we designated as the purely positive class (2,079 individuals) and which accounted for 76.4% of the data. The second class had high scores on Factor 1 and low scores on Factor 2, which we designated as the discriminatorily positive class (554 individuals) and which accounted for 20.4% of the data. The third class scored low on both factors, which we designated as the negative class (87 individuals) and which accounted for 3.2% of the data.

**Figure 4:**
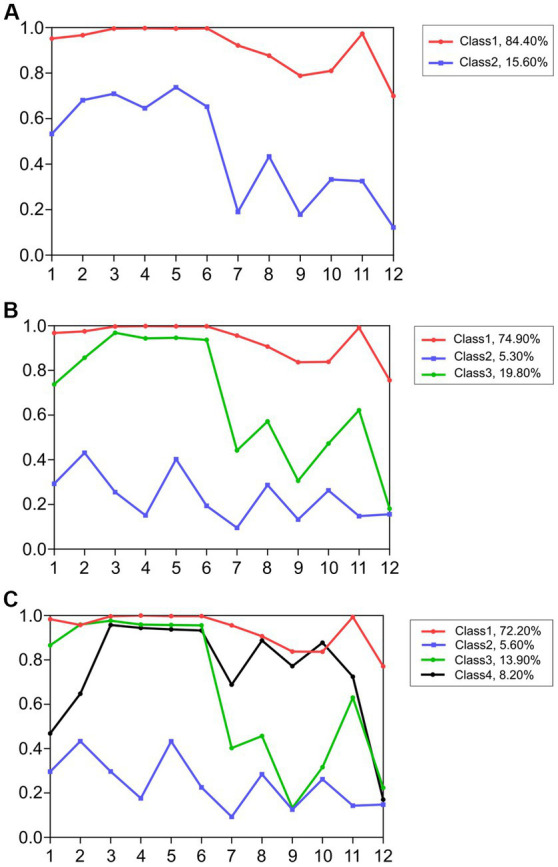
**(A)** Conditional probability diagrams of the two-class model (TAHS-G). **(B)** Conditional probability diagrams of the three-class model (TAHS-G). **(C)** Conditional probability diagrams of the four-class model (TAHS-G).

Figures 4A–C display the conditional probability diagrams of two to four classes of attitudes toward gay men. Similar to the findings regarding lesbians, the third class was more explanatory than the other classes. The purely positive class accounted for 74.9% of the data (2,036 individuals). The discriminatorily positive class accounted for 19.8% of the data (540 individuals), and the negative class accounted for 5.3% of the data (144 individuals).

## Discussion

Guo (2023) rethought how social culture (patriarchy and heterosexualism) influences attitudes toward homosexuality from a multiple sexual orientation perspective. As such, they developed the two-factor (*prejudice against lesbians/gay men* and *preference for heterosexuality*) and three-class (purely positive, discriminatorily positive, and negative classes) model of attitudes toward homosexuality, which introduced the *preference for heterosexuality* into psychometrics and drew attention to the discriminatorily positive attitude. To improve the applicability of the two-factor and three-class model and avoid potential measurement bias, we focused on specific subgroups—lesbians and gay men—and validated the consistency of the two-factor and three-class model of attitudes toward lesbians and gay men.

Additionally, we developed the TAHS-L and TAHS-G, which have acceptable reliability and validity. Previous studies have suggested that measurement invariance determines whether a set of indicators measures the same construct between different groups; if not, the results of group comparisons are meaningless, and the intergroup differences can be attributed to the measurement (Kline, 2011; Millsap, 2011). Thus, we assessed the TAHS-L’s and TAHS-G’s measurement invariance in terms of sex. Partial measurement invariance was obtained across males and females in both the TAHS-L and TAHS-G. Therefore, the TAHS-L/G can be used to compare intergroup differences in attitudes toward lesbians and gay men.

Compared with *prejudice against lesbians/gay men*—a negative attitude based on the opposites between groups—the *preference for heterosexuality* is a structurally and institutionally negative attitude influenced by social status. The implications of recognizing a *preference for heterosexuality* are noteworthy from the perspectives of social environment and human rights. The presence of a *preference for heterosexuality* in attitudes toward lesbians and gay men may lead to biased heteronormative assumptions, such as asking a cisgender woman, “Do you have a boyfriend?” Individuals who are aware of this factor are more likely than others to normalize diverse kinds of sexual identity and relationship status, which, in turn, helps build an inclusive environment bottom-up. In addition, institutions should acknowledge how the *preference for heterosexuality* is reflected in existing laws and policies and constantly maintains the hierarchical structure of rights between heterosexual people and sexual minorities. Even in countries with anti-discrimination laws, lesbians and gay men are widely disfranchised through limits imposed on the fundamental rights historically reserved for heterosexuals, such as civil union, marriage, and parenthood (Dotti Sani and Quaranta, 2022).

The ambivalent sexism theory (Glick and Fiske, 1996) suggests that hostile sexism and benevolent sexism are complementary rather than competing attitudes, meaning that an individual might simultaneously degrade a woman for deviating from her expected role and praise a woman for conforming to traditional gender stereotypes. Similarly, the three-class model of the TAHS-L and TAHS-G extends the theory of ambivalence to attitudes toward lesbians and gay men by identifying discriminatorily positive attitudes from a negative–positive unidimensional structure. People who hold such an attitude may not exhibit radical actions, such as physical violence toward or verbal harassment of lesbians or gay men, but rather pity them, thus constantly reinforcing the dominant status of heterosexism. In addition, the discriminatorily positive attitude could account for veiled homophobia in people’s daily lives. Veiled homophobia was proposed by Lyonga (2021) in a framework analyzing negative attitudes toward homosexuality that describes a sort of prejudice against lesbians and gay men masked by other justifiable forms.

However, we validated the aforementioned attitude toward lesbians and gay men as an independent typology rather than a subcategory under the synthetical concept of homophobia. In practice, the three-class model of attitudes offers potential explanations for the inconsistent efficacy in existing interventions targeting negative attitudes toward lesbians and gay men (Hodson et al., 2009; Rye and Meaney, 2009; Cramwinckel et al., 2021). Negative and discriminatorily positive attitudes are different and may undermine the well-being of lesbians and gay men through various mechanisms. Thus, unique intervention strategies for each attitude class may be needed to achieve an attitudinal shift to the purely positive attitude.

Of note, although discriminatorily positive attitudes are less apparent than negative or purely positive attitudes, they are not implicit. Implicit attitudes are habituated and automated, and they form a default attitudinal response when individuals encounter something that they deem atypical (Fazio and Towles-Schwen, 1999). The associative-propositional evaluation model—as modified by Bohner and Dickel (2011)—suggests that implicit attitudes are measured through the association activation process after information input and that explicit attitudes are measured through the proposition confirmation process after information input (metacognitive process). The former represents the activation of the attitude evaluating connection—regardless of whether the activating attitude is correct—while the latter relies on the subjective validation of the proposed attitude’s correctness. Unlike implicit attitudes, attitudes regarding the *preference for heterosexuality* rely on subjective validation based on different components of attitudes. Therefore, discriminatorily positive attitudes are nonobvious yet explicit, and their nonobvious characteristics result from their misclassification as positive attitudes and from the underestimation of their effects.

Although this study extends the targets of the two-factor and three-class models of attitudes toward lesbians and gay men, it has several limitations. First, we tested the TAHS-L and TAHS-G exclusively among subjects aged 18–40 years. Future studies would benefit from validating the measurement among a population with a broader age range. Second, the current study did not comprehensively examine the determinants of *prejudice against lesbians/gay men* and *preference for heterosexuality* in the attitudinal structure, nor was the influence of each class (i.e., purely positive, discriminatorily positive, and negative classes) on attitude analyzed in depth. Subsequent research can investigate the mechanisms by which the two-factor structure determines the cognitive and behavioral differences among individuals with different attitude classes during social interactions with lesbians or gay men. Third, we mainly focused on the factors and classes of explicit attitudes toward lesbians and gay men. However, whether a similar attitudinal structure exists in implicit attitudes toward lesbians and gay men remains to be examined.

## Conclusion

Based on a two-factor and three-class model of attitudes toward homosexuality, the present study developed two scales: a Two-factor Attitudes toward Lesbians Scale (TAHS-L) and a Two-factor Attitudes toward Gay Men Scale (TAHS-G). Each scale contains 12 items—six items each for Factor 1 (*prejudice against homosexuality*) and Factor 2 (*preference for heterosexuality*). The TAHS-L and TAHS-G have acceptable reliability and validity and partial measurement invariance for sex. Additionally, these scales can divide participants into three classes depending on whether they have purely positive, discriminatorily positive, or negative attitudes toward lesbians and gay men.

## Data availability statement

The raw data supporting the conclusions of this article will be made available by the authors, without undue reservation.

## Ethics statement

Ethical review and approval was not required for the study on human participants in accordance with the local legislation and institutional requirements. The patients/participants provided their written informed consent to participate in this study.

## Author contributions

LG contributed to developing the research design, collecting data, conducting data analyses, writing up the research in Chinese and revising the translated manuscript. SF contributed to collecting data, writing up the research in Chinese and improving the write-up. HW contributed to guiding the research design and revising the manuscript both before and after it was translated into English. All authors contributed to the article and approved the submitted version.

## Conflict of interest

The authors declare that the research was conducted in the absence of any commercial or financial relationships that could be construed as a potential conflict of interest.

## Publisher’s note

All claims expressed in this article are solely those of the authors and do not necessarily represent those of their affiliated organizations, or those of the publisher, the editors and the reviewers. Any product that may be evaluated in this article, or claim that may be made by its manufacturer, is not guaranteed or endorsed by the publisher.
